# Diagnosis of Food Protein-Induced Enteropathy Based on Gastrointestinal Mucosal Pathology before and after Elimination Diet Therapy: A Case Report

**DOI:** 10.3390/pediatric14030045

**Published:** 2022-09-19

**Authors:** Toshihiko Kakiuchi, Rie Furukawa

**Affiliations:** Department of Pediatrics, Faculty of Medicine, Saga University, 5-1-1 Nabeshima, Saga 849-8501, Japan

**Keywords:** food protein-induced enteropathy, food protein-induced enterocolitis syndrome, oral food challenge, esophagogastroduodenoscopy, pathology

## Abstract

We describe the case of a 1-year-old girl with food protein-induced enteropathy (FPE) that was difficult to diagnose. She was referred to our hospital with a 3-month history of diarrhea, vomiting, and weight loss. Although her diarrhea improved after a few days of fasting, oral intake of elemental diets, formula milk, or rice porridge resulted in repeated relapses. The serum IgE level was 1028 IU/mL, and radioallergosorbent tests were positive for milk, casein, alpha-lactalbumin, and other allergens. A histopathology of the duodenal mucosa revealed loss of mucosal villous structure, crypt hyperplasia, crypt apoptosis, and lymphocyte and eosinophil infiltration (<20 eos/hpf) into the lamina propria. After prednisolone (PSL) therapy and the complete removal of cows’ milk and chicken eggs from her diet, the patient’s diarrhea disappeared. Five months after discontinuing oral PSL and complete removal of cows’ milk and chicken eggs, the duodenum exhibited normal mucosal villous structure and well-differentiated ducts. No abnormalities were observed in the egg rechallenge; however, diarrhea recurred after the cows’ milk rechallenge. Thus, histopathologic examination of the gastrointestinal mucosa is useful for diagnosing FPE similar to oral food challenges, and re-evaluation after elimination diet therapy may be beneficial to rule out other diseases.

## 1. Introduction

Non-immunoglobulin E (IgE)-mediated gastrointestinal food allergies (non-IgE-GIFAs) and eosinophil gastrointestinal disorders (EGIDs) are collectively referred to as gastrointestinal allergies. Gastrointestinal allergies are allergic reactions with gastrointestinal symptoms and are classified into three types: IgE-dependent, non-IgE-dependent, and mixed with both properties. Normal immediate food allergies are IgE-dependent, non-IgE-GIFAs are not IgE-dependent, and EGIDs belong to the mixed gastrointestinal allergy group [1,2,3,4].

Non-IgE-GIFAs are classified as food protein-induced enterocolitis syndrome (FPIES), food protein-induced allergic proctocolitis (FPIAP), and food protein-induced enteropathy (FPE) [5,6,7]. FPIES can be subclassified into chronic FPIES that develops by ingesting daily allergens, which are common in Japan and South Korea; classical type in which the specific IgE is not detected; and atypical type in which the specific IgE is detected [1,2]. There are no clear diagnostic criteria for FPIAP and FPE [5].

Herein, we describe an infant case of FPE that was difficult to diagnose. Improvement in the gastrointestinal mucosal pathology after removal of the causative food, based on esophagogastroduodenoscopy (EGD) findings, facilitated the diagnosis.

## 2. Case Report

A 1-year-old girl was referred to our hospital with a 3-month history of diarrhea and weight loss. She was fed a mixed diet (breast and formula milk) from birth, beginning with rice and soybeans at 6 months and progressing to cows’ milk, wheat, and chicken eggs (yolk and white) at 8 months. She did not develop anaphylactic symptoms with formula milk and cows’ milk intake. There were no accompanying signs and symptoms related to other organs, such as mild eczema, skin dryness, or frequent upper respiratory tract infection due to cows’ milk allergy. Neither she nor anyone in her family had allergic diseases, such as atopic dermatitis or asthma. At 9 months of age, she developed vomiting, diarrhea, and anorexia; her previous diagnosis was infectious gastroenteritis and postenteritis syndrome. One month before her transfer to our hospital, she was admitted to the previous hospital because her symptoms did not improve for two months, and her weight loss progressed. During the 2 months prior to her admission, her diet was unrestricted. Although her vomiting improved after a few days of fasting, oral intake of elemental diets, formula milk, or rice porridge resulted in repeated diarrheas. Upon admission to our hospital, her physical examination revealed the following: height, 69.0 cm (standard deviation [SD] −2.2); bodyweight, 6641 g (SD −2.8), 999 g lesser than the weight measured 3 months ago; body temperature, 37.1 °C; heart rate, 96 beats per minute; and blood pressure, 88/50 mmHg. Her blood test results were as follows: white blood cell count, 16,900/mL (normal range [NR]: 7000–15,000); eosinophil percentage, 2.0% (NR: <5.6); hemoglobin, 9.9 g/dL (NR: 13.7–16.8); and platelet count, 679 × 10^3^ cells/mL. Her laboratory test findings were as follows: total protein, 6.4 g/dL (NR: 6.8–8.1); albumin, 3.9 g/dL (NR: 4.1–5.1); aspartate aminotransferase, 35 IU/L (NR: 20–45); alanine aminotransferase, 17 IU/L (NR: 4–24); blood urea nitrogen, 7.4 mg/dL (NR: 8–20); C-reactive protein, 0.47 mg/dL (NR: <0.03); sodium, 135 mEq/L (NR: 137–147); potassium, 3.6 mEq/L (NR3.6–5.2); bicarbonate, 19.1 mmol/L (NR: 21.0–27.0); and lactic acid, 21 mg/dL (NR: 4–16). At this time, a sufficient number of calories, amino acids, and fat was being administered intravenously. The serum IgE level was 1028 IU/mL (NR < 30), and radioallergosorbent tests were positive for milk (63.7 UA/mL class 5), casein (9.8 UA/mL class 3), alpha-lactalbumin (51.6 UA/mL class 5), egg white (58.8 UA/mL class 5), egg yolk (10.8 UA/mL class 3), ovomucoid (13.2 UA/mL class 3), wheat (14.5 UA/mL class 3), and soybean (1.3 UA/mL class 2). Skin prick tests were not performed to confirm the results of specific IgE test. Immunoglobulin (Ig) levels (IgG, 954 [NR 357–989] and IgM, 89 [NR 29–190]) and lymphocyte subset ratios were normal. Fecal eosinophil tests were negative, and no infection was detected in stool culture tests. Stool adenovirus, rotavirus, and norovirus antigen tests were negative.

**Figure 1:**
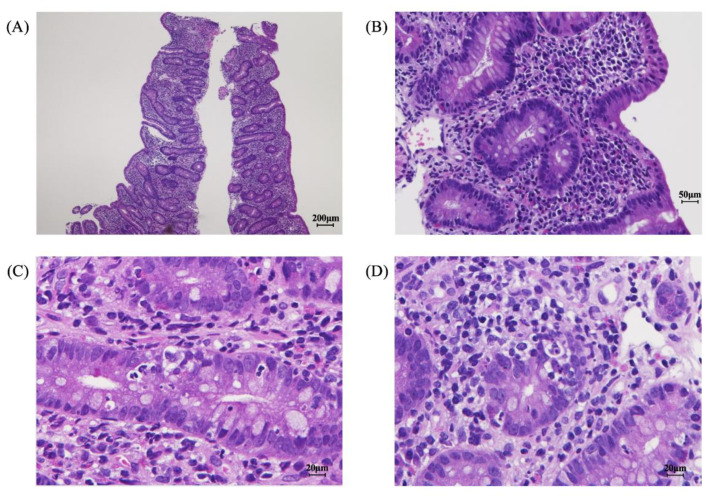
Pathologic findings in the gastrointestinal mucosa at diagnosis. Loss of mucosal villous structure, crypt hyperplasia (**A**,**B**), crypt apoptosis (**C**), and lymphocyte and eosinophil infiltration into the lamina propria were observed (**D**).

Imaging and endoscopy examinations were performed to determine the cause of the prolonged diarrhea. Abdominal computed tomography and ultrasound examinations revealed no abnormalities in the small intestine; the large intestine was dilated but no wall thickening was observed, suggesting nonspecific enteritis. An EGD showed no macroscopic abnormalities from the esophagus to the duodenum. However, histopathology of the duodenum mucosa revealed the loss of mucosal villous structure, crypt hyperplasia (Figure 1A,B), crypt apoptosis (Figure 1C), and lymphocyte and eosinophil infiltration (<20 eos/hpf) into the lamina propria, with partially formed crypt abscesses (Figure 1D). Total colonoscopy showed no abnormal macroscopic findings; however, histopathology of the colonic mucosa revealed erosion of the mucous membranes; cryptitis with decreased goblet cells, fibrosis, plasma cells, and lymphocytes; and some eosinophil infiltration (<20 eos/hpf) into the lamina propria. These findings were similar to the findings in the duodenum mucosa.

**Figure 2:**
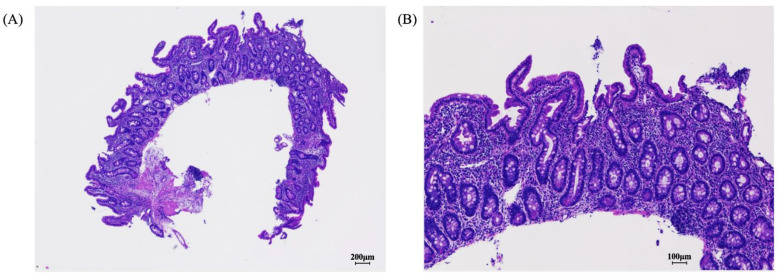
After 5 months of elimination diet therapy, pathologic findings in the gastrointestinal mucosa showed restored normal mucosal villous structure with well-differentiated ductal arrangement (**A**,**B**).

Autoimmune enteropathy and inflammatory bowel disease (IBD) were suspected; therefore, additional tests were performed. Antiautoimmune enteropathy-related 75 kDa antigen [8] and anti-villin antibodies [9] were absent and no significant pathogenic variants were found in 25 genes (covered by public insurance in Japan) related to monogenic IBD and immunodeficiency, polyendocrinopathy, enteropathy X-linked (IPEX) syndrome. EGID was considered in the differential diagnosis, but a diagnosis of FPE was finally suspected based on the characteristics of the gastrointestinal mucosa, such as atrophy of small intestinal mucosal villi, crypt hyperplasia, eosinophil infiltration below the EGID diagnostic criteria (<20 eos/hpf), and lymphocyte infiltration. Her peripheral blood eosinophil count was also not elevated. Chronic atypical (positive for specific IgE) non-IgE-GIFA was suspected (7). Her diarrhea disappeared after prednisolone (PSL) therapy (1 mg/kg) and the complete removal of formula milk, cows’ milk, and chicken eggs from her diet. No relapse occurred even after discontinuing PSL one month later. Adverse effects due to long-term treatment with PSL, such as hypertension, did not occur. Five months after discontinuing oral PSL and the complete removal of formula milk, cows’ milk, and chicken eggs, duodenal histopathology revealed normal mucosal villous structure and well-differentiated ductal arrangement (Figure 2A,B). Three months after the second EGD, she resumed eating chicken eggs without experiencing gastrointestinal symptoms such as diarrhea and vomiting; however, 3 months later, her symptoms, including abdominal pain and diarrhea, relapsed within days of resuming cows’ milk consumption. Based on the abovementioned findings, FPE was diagnosed, with cows’ milk suspected to be the allergen source. At 2 years and 3 months, her height had improved to 81.5 cm (SD −1.4) and her weight to 11.4 kg (SD +0.33) without the occurrence of gastrointestinal symptoms.

## 3. Discussion

Diagnosis of non-IgE-GIFAs is difficult for the following reasons: (1) non-IgE-GIFAs are mainly caused by cows’ milk; however, rice, soybeans, wheat, chicken eggs, allergens in maternal diet transmitted through the consumption of breast milk, and other allergens can also cause non-IgE-GIFAs; (2) the causative food cannot be estimated from the specific IgE antibody, as non-IgE-GIFAs is not IgE-dependent; (3) 30% of non-IgE-GIFAs are also positive for specific IgE similar to IgE-dependent immediate food allergies; and (4) no clear diagnostic criteria for FPIAP and FPE exist. In the present case, the specific IgE antibodies were also positive for foods other than milk. The gastrointestinal mucosal findings obtained using EGD before and after elimination diet therapy were helpful in making the diagnosis.

Non-IgE-GIFAs may not be fully diagnosed [6], and the actual diagnosis relies on the recognition of symptom patterns in FPIAP and FPIES and biopsies in FPE. No accepted diagnostic criteria are available for FPE and FPIAP. However, some elements routinely used in clinical practice support the diagnosis of these diseases according to international consensus guidelines [1]. FPE presents in young infants (<9 months) with typical symptoms of vomiting and intestinal malabsorption. In addition, a biopsy suggestive of FPE helps confirm this diagnosis [5,10]. In FPIAP, the causative association between rectal bleeding and antigenic food must be established with a resolution of symptoms upon elimination of the offending foods and, preferably, with the documented recurrence of symptoms when foods are re-introduced [11]. Furthermore, other causes of hematochezia, such as anal fissures, must be excluded [7].

FPE causes diarrhea that lasts for more than 2 weeks, leading to poor weight gain and stunted growth due to digestive and absorptive disorders. The pathologic findings for FPE are well known, and histologic findings such as atrophy of small intestinal mucosal villi, crypt hyperplasia, and lymphocyte infiltration are useful for diagnosis. These features were observed in the present case. In contrast, EGIDs are characterized by extensive eosinophilic inflammation and mast cell infiltration [12]. In the clinical setting, non-IgE-GIFAs may be misdiagnosed because this is primarily a clinical diagnosis [13], and the symptoms do not appear immediately after ingesting the causative food antigen. However, in the present case, histopathologic evaluation of the gastrointestinal mucosa helped establish her condition, and the restoration of villous structures after milk elimination diet therapy confirmed the diagnosis. The elimination diet was very helpful in distinguishing allergic diseases such as non-IgE-GIFAs and EGIDs from nonallergic diseases such as monogenic IBD and IPEX syndrome. This is because the elimination diet alone cannot be expected to improve disease findings in nonallergic diseases. Villous atrophy is often also observed in celiac disease and microscopic enterocolitis; however, in the present case, abnormal findings were not limited to the duodenum, which is characteristic of celiac disease, and in microscopic enteritis, diarrhea did not improve dramatically with only short-term steroids and an elimination diet. Thus, celiac disease and microscopic enteritis were ruled out.

In allergic diseases, removal of the causative antigen facilitates both diagnosis and treatment. Elimination diet therapies have not been established for non-IgE-GIFAs; therefore, it is difficult to assess improvements in gastrointestinal symptoms with these therapies. Elimination of the offending food significantly improves emesis and diarrhea within a few hours in patients with acute FPIES and within days in those with chronic FPIES. Visible blood in the stool resolves within a few days in patients with FPIAP. However, in patients with FPE, symptoms usually resolve in 1–4 weeks [5]. The variable time to recovery makes it difficult for clinicians to assess the effectiveness of the elimination diet. Moreover, oral food challenges (OFCs) in allergic disorders involve ethical issues, and universal agreement on the optimal method to perform OFCs in non-IgE-GIFAs is currently lacking [14]. OFCs represent the gold standard in confirming the diagnosis of FPIES and are particularly important in preventing the misdiagnosis of other common gastrointestinal diseases in infancy. However, OFC for suspected FPIES is considered a high-risk procedure [7]. As with OFC, which is the gold standard for the diagnosis of non-IgE-GIFAs, endoscopic pathologic findings before and after the administration of an elimination diet may also be useful in the absence of an established method for non-IgE-GIFAs at present.

With regard to the present case, we should be careful about her elevated IgE levels when diagnosing FPE. Approximately 30% of non-IgE-GIFAs are known to be positive for specific IgE [1]. In the present case, the patient’s OFC was unresponsive to chicken eggs but reacted to cows’ milk days rather than hours after ingestion, implying a non-IgE-dependent rather than an immediate allergic reaction. In addition, her FPE did not respond to the elimination diets and hydrolyzed milk at the previous hospital, and she experienced repeated diarrhea symptoms. This was thought to be because the period until the start of formula milk administration was too short, i.e., only a few days.

If a restricted diet method has not been established, pathologic evaluation by EGD in infants may be necessary for distinguishing between allergic and nonallergic diseases. Even though EGD is invasive, the procedure may be necessary for eliminating unnecessary testing and surgical interventions [15]. In conclusion, the pathologic findings of the gastrointestinal mucosa are useful for diagnosing FPE, and re-evaluation after elimination diet therapy is effective in differentiating FPE from other diseases.

## Data Availability

Not applicable.
